# Control of Innate Immunity by Sialic Acids in the Nervous Tissue

**DOI:** 10.3390/ijms21155494

**Published:** 2020-07-31

**Authors:** Huan Liao, Christine Klaus, Harald Neumann

**Affiliations:** Neural Regeneration, Institute of Reconstructive Neurobiology, Medical Faculty and University Hospital of Bonn, University of Bonn, Venusberg-Campus 1, D-53127 Bonn, Germany; s4huliao@uni-bonn.de (H.L.); christine.klaus@uni-bonn.de (C.K.)

**Keywords:** sialic acid, SIGLEC, complement, microglia, desialylation, neuroinflammation, neurodegeneration, aging

## Abstract

Sialic acids (Sias) are the most abundant terminal sugar residues of glycoproteins and glycolipids on the surface of mammalian cells. The nervous tissue is the organ with the highest expression level of Sias. The ‘sialylation’ of glycoconjugates is performed via sialyltransferases, whereas ‘desialylation’ is done by sialidases or is a possible consequence of oxidative damage. Sialic acid residues on the neural cell surfaces inhibit complement and microglial activation, as well as phagocytosis of the underlying structures, via binding to (i) complement factor H (CFH) or (ii) sialic acid-binding immunoglobulin-like lectin (SIGLEC) receptors. In contrast, activated microglial cells show sialidase activity that desialylates both microglia and neurons, and further stimulates innate immunity via microglia and complement activation. The desialylation conveys neurons to become susceptible to phagocytosis, as well as triggers a microglial phagocytosis-associated oxidative burst and inflammation. Dysfunctions of the ‘Sia–SIGLEC’ and/or ‘Sia–complement’ axes often lead to neurological diseases. Thus, Sias on glycoconjugates of the intact glycocalyx and its desialylation are major regulators of neuroinflammation.

## 1. Introduction

Glycosylation extends the functional diversity of proteins and lipids tremendously. It is one of the most complicated posttranslational modifications, which is added in the endoplasmic reticulum after the folding of glycoproteins or build-up of glycolipids. Thereby, proteins and lipids are elongated by various saccharides leading to high glycan diversity. The added saccharides mainly contribute new functional properties related to complex cell-cell and cell-matrix interactions [1]. Here, sialic acids (Sias) play a unique role. While Sias are displayed on the glycocalyx of few pathogens, such as *Campylobacter jejuni* and *Neisseria* spp. [2], they are abundantly present on most extracellular proteins and lipids in mammals. Sias mostly form terminal caps on the glycoconjugates of the mammalian glycocalyx. In addition, mammals have a huge repertoire of immune receptors to recognize Sias in conjunction with the underlying structures. Therefore, Sias have been introduced as essential determinants of self-recognition by the immune system [3]. Particularly, the innate immune system has developed several receptors for recognition of Sias on glycoconjugates as self-patterns. Both complement regulator factor H (CFH) and Sia-binding immunoglobulin-like lectins (SIGLECS) have been recognized as sensors of Sia-containing self-patterns [4,5].

The complement cascade is part of the innate immune system. It is the first line of defense against pathogens, but also plays a pivotal part in tissue homeostasis. Thus, it also contributes to several processes in development, aging and diseases [4,6,7]. In the central nervous system (CNS) several components of the classical complement system, including complement component 1 (C1), complement component 3 (C3), complement receptor 3 (CR3) and complement system-associated receptors (e.g., triggering receptor expressed on myeloid cells 2—TREM2), have been shown to contribute to axonal pruning and fine-tuning of synaptic connections during development of neurons. The formation and activation of a C3 convertase is a key step in the complement cascade. To avoid host tissue damage caused by unnecessary activation of the complement system, the activation of C3 is tightly regulated by distinct triggers from the classical, alternative or lectin complement pathways. Furthermore, the C3 convertase is modulated by the fluid-phase inhibitor CFH, as well as the complement activator properdin. The function and localization of CFH is dependent on its selective binding to α2,3-linked sialoglycans with its most C-terminal domain [8]. This self-recognition process of CFH binding to Sias helps to avoid indiscriminate activation of C3 and inappropriate host tissue damage caused by the alternative complement cascade [4].

SIGLECS are Sia-recognizing receptors that belong to the family of transmembrane Ig-type lectins. Lectins are proteins or glycoproteins that bind to glycans. SIGLECS directly interact with sialoglycans due to their Sia-binding domain in the extracellular part. Thus, SIGLECS are prototypic receptors for self-recognition [5,9]. While most SIGLECS are specifically expressed on innate and adaptive immune cells, a few SIGLECS are detected on other cell types such as SIGLEC-4/ myelin-associated glycoprotein (MAG) on oligodendrocytes [9] or SIGLEC-11 on ovarial stromal cells [10]. Humans have 15 members of the SIGLEC family, while mice only have nine members. Each member has a different, but partially overlapping, binding specificity to sialoglycans [5]. Most SIGLECS have intracellular immunoreceptor tyrosine-based inhibitory motifs (ITIMs) that counteract the activation of immune cells via immunoreceptor tyrosine-based activation motif (ITAM) signaling coming from ligand recognition of associated receptors. Hence, SIGLECS contribute to control of immunity and play a critical role in the avoidance of detrimental immune reactions by dampening unwanted tissue inflammation-induced damage [11]. Particularly, chronic inflammation occurs as one of the hallmarks of aging [12] and the intact interaction between sialic acid residues and SIGLEC-E has been recognized to play an important role in the maintenance of the normal lifespan of mice. Genetic deletion of *Siglec-E* in mice was leading to increased reactive oxygen species production in several tissues and caused inflammation, oxidative damage, grey hairs and reduced life span [13]. This finding gave strong evidence that the ‘Sia-SIGLEC’ axis plays a central role in maintaining innate immune and tissue homeostasis in the long term and suggests that altered sialylation related to aging might trigger neuroinflammatory processes.

This review summarizes recent literature focusing on the self-recognition of Sias of glycoconjugates via the complement system and the SIGLECS in the neural tissue, as well as the effects of an altered recognition on neuroinflammatory processes and its involvement in neurological diseases.

## 2. Brain Innate Immunity

Microglia are the key innate immune cells of the CNS. They play a pivotal role in brain development, maturation and homeostasis, but also could transform into macrophage-like cells with a professional innate immune defense function. Microglial cells scan the brain parenchyma continuously by their processes to sense the microenvironment and react appropriately [14]. Based on this input, microglia switch between the immunological phenotypes of silence and activation. While an appropriate response of microglia contributes to brain tissue homeostasis and repair, an inappropriate response can lead to neural tissue damage and diseases, such as neurodegeneration [15]. Interestingly, an inappropriate response could be either too much activation leading to oxidative damage or paralysis leading to insufficient phagocytic removal of debris or aggregated proteins.

Recently, it has been proposed that disease-associated microglia (DAM), characterized by a defined transcriptome profile, are major players in neurodegenerative disorders [16,17]. A comprehensive single-cell RNA sequencing (RNA-seq) analysis identified DAM under neurodegenerative conditions as a unique subset of microglia that developed in a *Trem2*-dependent manner [16]. This DAM subset showed upregulation of well-known microglial markers in conjunction with genes related to lysosomal, phagocytic, and lipid metabolism pathways, such as *Iba1*, while genes related to homeostasis were downregulated [17,18]. Importantly, it was suggested that DAM sense the CNS damage early and protect the brain from neurodegeneration [17], although the full consequences of the DAM subset in distinct disease processes remained not fully understood. However, it was becoming clear that innate immune recognition receptors of microglia signaling via ITAM, particularly TREM2, are the key molecules for microglia to sense damage and initiate a proper immune response [19].

**Figure 1:**
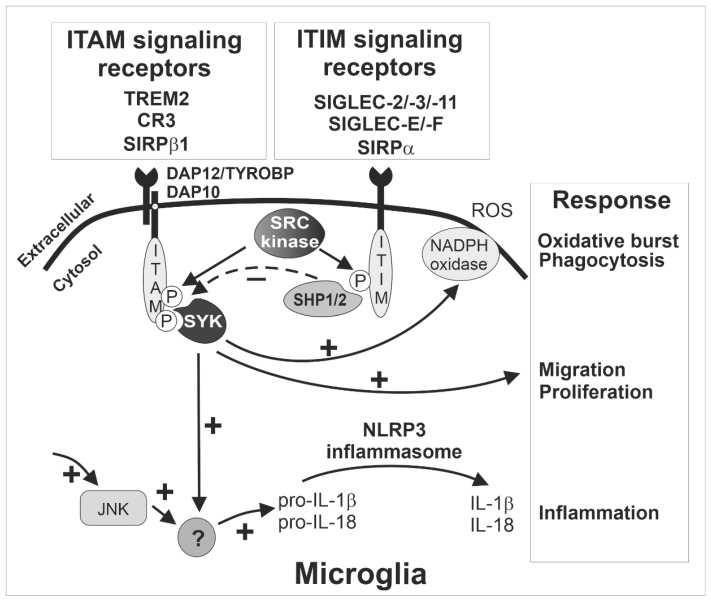
The ITAM- and ITIM-signaling receptors sense the microenvironment and determine the response of microglia. Inhibitory SIGLEC receptors such as SIGLEC-2/-3/-11 (human) and SIGLEC-E/-F (mouse) as well as other immunoreceptor tyrosine-based inhibition motif (ITIM)-signaling receptors (e.g., SIRPα) recruit and activate SHP1/2, which can in turn terminate intracellular signals emanating from immunoreceptor tyrosine-based activation motif (ITAM)-signaling receptors via their intrinsic phosphatase activity. The ITIM signaling pathway inhibits SYK activation (dotted ‘-‘ arrow) and thus prevents several responses of microglia (‘+’ arrows) including phagocytosis, oxidative burst, migration, proliferation, and inflammation. The exact signaling cascade of the counterbalanced ITAM- and ITIM-mediated response is not fully understood, but several cell membrane receptors converge on the ITAM/ITIM-response to elicit diverging signals via several intracellular second messenger pathways (‘+’ arrows). Interestingly, ITIM signaling via inhibition of SYK also down-regulates inflammasome activation, since both SYK- and JNK-dependent pathways are required for inflammasome activation. CR3, complement receptor 3 as heterodimer of CD11b/ITGAM and CD18/ITGB2; HSCT/DAP10, hematopoietic cell signal transducer; IL, interleukin; ITAM, immunoreceptor tyrosine-based activation motif; ITIM, immunoreceptor tyrosine-based inhibition motif; JNK, C-Jun NH2-terminal protein kinase; NADPH, nicotinamide adenine dinucleotide phosphate; NLRP3, nucleotide-binding oligomerization domain-leucine-rich repeats containing pyrin domain 3; P, phosphate; ROS, reactive oxygen species; SHP1/2, Src homology region 2 domain-containing phosphatase-1/2, also named PTPN6/PTPN11 gene; SIGLEC, sialic acid-binding immunoglobulin type lectin; SIRP, signal regulatory protein; SYK, spleen tyrosine kinase; TREM2, triggering receptor expressed on myeloid cells 2; TYROBP/DAP12, TYRO protein tyrosine kinase binding protein/DNAX-activating protein of 12 kDa.

These ITAM-signaling innate immune receptors of microglia play a critical role in activation and phagocytosis. After ligand binding, the recognition receptor associates with the ITAM-bearing adaptor protein DNAX-activating protein of 12 kDa (TYROBP/DAP12; Figure 1). Subsequently, the intracellular ITAMs are phosphorylated by members of the SRC kinase family, leading to the formation of docking sites for spleen tyrosine kinases (SYK). The activation of SYK initiates a series of downstream signaling cascades, resulting in microglia activation and phagocytosis [19] (Figure 1). As mentioned above, the inhibitory ITIM signaling of SIGLECS counteract the activatory effects of DAP12 via dephosphorylation of the ITAMs.

TREM2 appears to be one of the most relevant ITAM-signaling receptors of microglia. TREM2 can recognize ligands from both bacteria and mammalian cells, such as lipooligosaccharides from *Neisseria gonorrhoeae* [20], lipopolysaccharides (LPS) from *E. coli* [21], human apolipoprotein E [22], and aggregated proteins such as amyloid-β [23]. It interacts with DAP12 to form the TREM2/DAP12 complex. Of note, this complex transmits ITAM signaling to regulate microglial activity, such as phagocytosis and cytokine production [24]. As mentioned before, accumulating evidence has demonstrated that TREM2/DAP12 signaling plays a major role in changing microglia from a homeostatic state to a disease-associated state called DAM. More importantly, variants of TREM2 with reduced ligand binding capacity are strong risk factors of Alzheimer’s disease (AD) [25,26] and frontotemporal dementia [27,28]. In addition, an integrative network-based approach displayed that DAP12/TYROBP is a key regulator of late-onset AD [29] and in another systems biology-based study, using a weighted gene co-expression network analysis, DAP12/TYROBP was among the hub genes of a preserved network that was strongly related to neurodegenerative diseases, but also to aging [30]. Furthermore, this gene regulatory network was enriched with a microglial signature. Moreover, loss-of-function mutations of either *TREM2* or *DAP12* are related to the development of a presenile dementia with bone cysts, a rare hereditary autosomal-recessive disease called Nasu–Hakola disease or polycystic lipomembranous osteodysplasia with sclerosing leukoencephalopathy [31,32]. Unexpectedly, this phenotype cannot be mimicked in mice since the deletion of *Trem2* was leading to an opposite phenotype with reduced loss of neurons during physiological aging [33]. In mice, it even appeared that *Trem2* had detrimental oxidative and neurodegenerative effects in the specific-pathogen-free housed wildtype mice, compared to *Trem2* knock-out mice that showed less age-related neuroinflammation and less loss of neurons [33]. Similarly, the deficiency of *Dap12* was neuroprotective in a mouse model of early AD [34]. Thus, the absence of TREM2-mediated ITAM signaling has been shown to contribute to insufficient removal of surplus neuronal connections during development, but also less clearance of still intact neurons during aging of mice, that were kept in an environment without pathological stimuli. However, in humans loss of TREM2/DAP12 is leading to lipid accumulation and neurodegeneration via a mechanism that is still unclear and difficult to fully recapitulate in mice.

Complement receptor 3 (CR3, also known as CD11b/CD18) is another microglial receptor mediating ITAM signals via TYROBP/DAP12. As mentioned before, microglia can target synapses [35] and play a critical role in axonal and synaptic pruning via the classical complement cascade [36], thus contributing to normal brain development [37]. The classical complement cascade is initiated by the complement component 1q (C1q) opsonization that triggers a cascade of different complement factors including the formation of the C3 convertase complex. The neuronal C1q/C3-opsonized structures can be removed via CR3-mediated phagocytosis of microglia during development, possibly involving ITAM signaling of DAP12 [36,38]. Likewise, the microglial innate immune receptor TREM2 that signals via ITAMs of DAP12 is also involved in synapse elimination and development of normal brain connectivity [39]. Inappropriate activation of CR3 signaling led to early synapse loss in an AD mouse model [40]. Interestingly, both mouse and human in vitro culture systems demonstrated that desialylated neurites can trigger their removal via CR3-mediated phagocytosis by co-cultured microglia or macrophages [41,42]. Thus, the sialylation of axons and dendrites appears to be an important determinant for preventing inappropriate elimination by microglia via CR3. However, it is unclear from this model whether the missing inhibition of the complement system via CFH, the lack of SIGLEC binding to Sias of glycoconjugates or even the uncovering of the Sia-underlying structures that might trigger complement opsonization are responsible for the CR3-mediated phagocytosis.

Accordingly, sialylation tightly regulates the brain innate immune system, particularly the complement system and the activation of the microglia via ITAM- and ITIM-signaling receptors and keeps the microglial function in a homeostasis status, while following appropriate immune responses.

## 3. Sialic Acids (Sias): Structure, Diversity, Biosynthesis Related Key Enzymes and Sialidases

Sias are nine carbon monosaccharides. They form the terminal caps on the glycocalyx of cell surfaces and secreted glycoproteins of vertebrates [43]. There are three main types of Sias in vertebrates, namely N-acetylneuraminic acid (Neu5Ac), N-glycolylneuraminic acid (Neu5Gc), and deaminoneuraminic acid (Kdn). While Neu5Ac is the most abundant Sia in humans, Neu5Gc and Kdn only are detected as trace amounts in human tissue, most likely derived from food sources [44,45,46,47]. The three major Sias are further subject to a number of modifications, such as methylation, sulfation, lactylation, acetylation, and lactonization and form a diverse family consisting of more than 50 members with different structures [48]. Sias are highly abundant on the surface of nervous and immune cells of mammals. For example, Sias have an estimated concentration of more than 100 mM at the surface of a lymphocyte [49]. However, the exact level of cell surface sialylation of human microglia or neurons is unclear.

**Figure 2:**
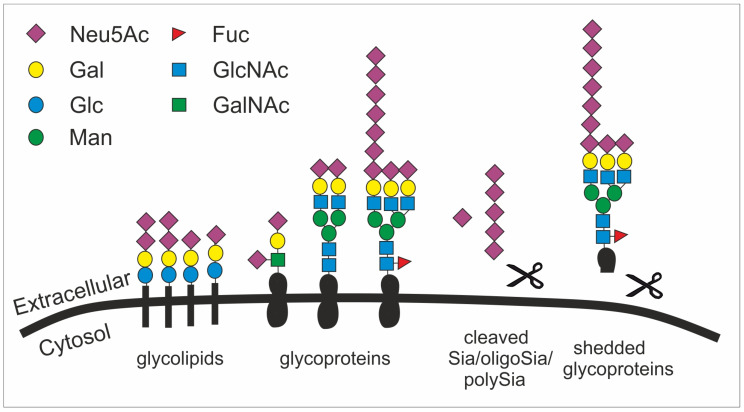
The structural diversity of the glycocalyx is fundament to many cellular processes and appears with the sialic acid subunit Neu5Ac in humans as terminal cap. Sialic acid is mainly found as terminal saccharide on the glycocalyx that is formed by glycolipids (e.g., gangliosides) as well as sialylated and polysialylated glycoproteins (e.g., polySia-NCAM). Glycoproteins can be shedded (here simplified by showing a scissor) and released as sialylated molecules (e.g., forming the mucus). Sialic acids (Sia), oligosialic acids (oligoSia) and polysialic acids (polySia) can be cleaved by sialidases (scissor) or oxidative processes (scissor) and trapped together with growth factors in the extracellular matrix. Neu5Ac, N-acetylneuraminic acid; Gal, galactose; Glc, glucose; Man, mannose; Fuc, fucose; GlcNAc, N-acetylglucosamine; GalNAc, N-acetylgalactosamine.

The biosynthesis of Sia takes place in the cytosol and involves four steps and three enzymes. Among the three enzymes, the bifunctional glucosamine (UDP-N-Acetyl)-2-epimerase/N-acetylmannosamine kinase (GNE) is one rate-limiting enzyme [50]. GNE participates in the initial two steps of Sia biosynthesis. After catalyzing the conversion from uridine diphosphate N-acetylglucosamine to N-acetyl-D-mannosamine (ManNAc), GNE further phosphorylates ManNAc to form N-acetyl-mannosamine 6-phosphate (ManNAc-6-P). ManNAc-6-P is then condensed and dephosphorylated to generate Neu5Ac by N-acetylneuraminic acid synthase and N-acetylneuraminic acid phosphatase [51]. The Neu5Ac is then conjugated with cytidine 5′-monophosphate (CMP) to form active CMP-Neu5Ac by the enzyme cytidine monophosphate N-acetylneuraminic acid synthetase in the nucleus [52]. Subsequently, sialyltransferases transfer Sias to various molecules terminated with a galactose (Gal), an *N*-acetylgalactosamine (GalNAc), or another Sia residue (Figure 2). This step leads to the biosynthesis of Sia-containing glycoconjugates and oligosaccharides [53]. There are 20 types of sialyltransferases in humans that conjugate the Sia molecule Neu5Ac to the acceptor sugar (Gal, GalNAc, Neu5Ac) using a defined linkage (α2,3-, α2,6-, α2,8-) between the Sia residue and the acceptor sugar. Moreover, Sias can form homopolymers with different degrees of polymerization via intersialyl linkages. For instance, polysialic acid (polySia) is a homopolymer of α2,8-linked Neu5Ac monomers with a degree of polymerization between 10 and around 200 [54]. Thus, a huge number of glycoconjugates can be generated.

Given their abundance and diversity on all vertebrate cell surfaces as the terminal cap, it becomes obvious that Sias fulfill multifarious roles, such as in microdomain formation [55], cell adhesion [56], tissue homeostasis [57], immune cell modulation [58], cell migration [59], chemokine sensing [60], and growth factor retention [61] (Figure 2). Particularly, the outermost position of Sias with multiple functional groups allows them to have interactions with other molecules, mainly glycoproteins called lectins. These interactions involve hydrogen bonds, salt bridges, and non-polar interactions. However, the affinity of such interactions between one protein (e.g., a lectin) and the respective glycan is relatively low and often requires multivariant binding between several glycans and protein molecules [62].

Interestingly, some bacteria can use this self-recognition system as camouflage by generating analogues of human sialoglycans. For example, α2.8-linked polySia is used by several bacteria as a neuro-invasive determinant [63,64] and thus could escape host immune attack via inactivating the complement system and silencing innate immune cells via SIGLEC-11. The polySia capsules of *Escherichia coli* K1 (*E. coli* K1) specifically restrict alternative complement pathway activation [65], while those of serogroup B *Neisseria meningitides* help the bacteria to escape elimination by normal human serum [66]. However, the mechanism of how polySia modulates the complement cascade is not fully understood yet. Furthermore, polySia is used by *E. coli* K1 for immune evasion by a second receptor-mediated mechanism: the human-specific pathogen *E. coli* K1 uses its polySia capsule as a protective molecular shield to escape immune defense by engaging SIGLEC-11 [67].

Sialidases/neuraminidases are glycoside hydrolase enzymes. They participate in the cleavage of terminal Sias from glycoproteins and glycolipids, a process named desialylation [68]. In mammalian cells, there are four types of sialidases, sialidase 1–4. Sialidase 1 (a lysosomal sialidase, also called N-acetyl-alpha-neuraminidase 1; NEU1) is encoded by the *NEU1* gene. It is present in both plasma membrane and lysosome. Sialidase 2 (a cytosolic sialidase, also called N-acetyl-alpha-neuraminidase 2; NEU2), encoded by the *NEU2* gene, can be found in the cytosol. Sialidase 3 (a membrane sialidase, also called N-acetyl-alpha-neuraminidase 3; NEU3) is encoded by the *NEU3* gene and localized in the plasma membrane, while sialidase 4 (N-acetyl-alpha-neuraminidase 4; NEU4) is encoded by the *NEU4* gene and displays on internal membranes. In the mouse brain, NEU1, NEU3 and NEU4 show similar expression patterns [69,70]. Moreover, these sialidases are active against different but overlapping sialylated glycoconjugates. Both NEU1 and NEU4 are active towards sialylated oligosaccharides, glycoproteins, and the gangliosides as glycolipids. However, NEU1 has negligible activity against gangliosides, which are the main substrates of NEU3 [70]. Growing evidence suggests that mammalian sialidases play an important role in the development and function of the CNS [54,68,70].

## 4. Sialic Acid (Sia)-Related Glycoconjugates, Expression Pattern and Function in the Brain

The majority of sialic acid in the mammalian brain is detected on gangliosides [71,72]. They carry around 75% of the total brain sialic acids [54,73,74]. Gangliosides have a common lipid core structure, but differ in the position and number of Sias that are linked to the lipid headgroup that is integrated into the membrane bilayer. Around 60 subtypes of gangliosides are known. The four major brain gangliosides are GM1, GD1a, GD1b, and GT1b. Gangliosides are essential for the development of the CNS [75] and in adulthood they help to maintain healthy cellular interactions. For instance, in *cis* interactions they are involved in the proper formation of lipid rafts, while in *trans* they interact with possible binding partners, such as SIGLECS. The ganglioside subtype GM1 has been suggested as acting in a neuroprotective way in the brain [76]. Importantly, multiple small-sized clinical trials have indicated a potential therapeutic effect of applied GM1 in various neurological diseases [76], although a well-powered and placebo-controlled trial has not been performed so far. The level and subtype of ganglioside expression varies during development. In the developing brain quite simple gangliosides are expressed (e.g., GM3, GD3), while more complex gangliosides, such as GD1a and GT1b, appear after birth [77,78]. In adults, gangliosides are differently distributed all over the brain [79,80,81].

Compared to other tissues, polysialylation is highly abundant in the nervous tissue. The sialyltransferases ST8Sia2 and ST8Sia4 are responsible for the polysialylation of glycoconjugates. Intriguingly, the embryonic brain has much higher levels of the α2,8-linked polySia than those detected in the adult brain. Besides, there is a decrease in neural sialyltransferase activity in post-mortem brain from AD patients compared with age-matched controls [82]. While 85% of α2,8-linked polySia is attached to the neural cell adhesion molecule (NCAM), approximately 15% of α2,8-linked polySia is present on other glycoconjugates [83] such as synaptic cell-adhesion molecule [84] and neuropilin-2 [85]. Some of these polysialylated glycoproteins are also present on microglia [86]. A recent study confirmed the presence of polysialylated NCAM on microglia by using the mouse microglial cell line Ra2. This study also showed that the polySia on the microglia cell surface was rapidly cleared by the exovesicular sialidase NEU1 upon lipopolysaccharides (LPS) challenge [86]. Interestingly, *Ncam* (-/-) microglia have Golgi-confined polySia and their carrier is neuropilin-2 [87]. Upon microglial activation by LPS, Golgi-localized polySia was translocated to the cell surface and completely depleted from the Golgi apparatus [87].

The expression pattern of polySia in the mouse brain has been well studied during development until adulthood. Polysialylation starts from embryonic day 9.5 and reaches peak level just before birth. At 8 weeks after birth, polySia expression is strongly reduced in most brain regions, but still present on synapses [61], in the neurogenic zones of the hippocampus and dental gyrus [88], and in the retina [89]. Therefore, adult brains still have polySia-NCAM in areas related to neurogenesis and neural plasticity, such as the hippocampus, as well as specialized nervous regions, such as the retina [61]. No clear information on the expression level of polySia during aging is available, but data indicate that the expression of polySia is reduced during aging [90].

Polysialylation in the nervous system plays a critical role in the modulation of innate immune functions, regulation of synaptogenesis, neurogenesis, cell proliferation and migration, axon guidance, fasciculation, learning, memory, and cell adhesion [54]. Additionally, polysialylation is involved in capturing neurotrophic factors, growth factors, neurotransmitters and ions, cytokines, chemokines, and transcription factors [61,91,92]. For example, brain-derived neurotrophic factor (BDNF) locally produced and retained in the extracellular matrix of the CNS is bound to polySia under normal conditions. However, it is released under inflammation conditions, when polySia on microglia is removed by the sialidase NEU1 [86].

## 5. Sialic Acid (Sia)-Related Signaling: Complement, SIGLECS and Other Receptors 

The complement system plays a key role in innate immune surveillance and homeostasis. It has three different pathways for activation, (1) the classical, (2) the lectin, and (3) the alternative pathway [4]. All three pathways have distinct initial triggers, but merge during C3 activation. Then, the inactive C3 protein is cleaved via the C3-convertase into the functional complement component 3a (C3a) and the complement component 3b (C3b). Whereas C3a mediates inflammation, C3b acts as an opsonin to tag any surface nearby for phagocytosis [93]. Of note, the alternative pathway may contribute to the other two pathways and appears to be active during up to 90 % of all complement activation events [94]. Unlike the other two pathways, the alternative pathway maintains a low level of activity during normal physiological conditions by spontaneous hydrolysis of an internal C3-thioester bond [4]. Thus, the alternative pathway needs a tight control to avoid inappropriate damage to host tissues. While properdin acts as a C3-convertase (C3bBb) stabilizer to amplify the alternative pathway, CFH negatively regulates the alternative pathway [95,96]. The CFH competes with complement factor B (CFB) that binds to C3b to form the C3 convertase (C3bBb). It also dissociates pre-existing C3-convertases (C3bBb) and helps complement factor I (CFI) to degrade C3b [97].

**Figure 3:**
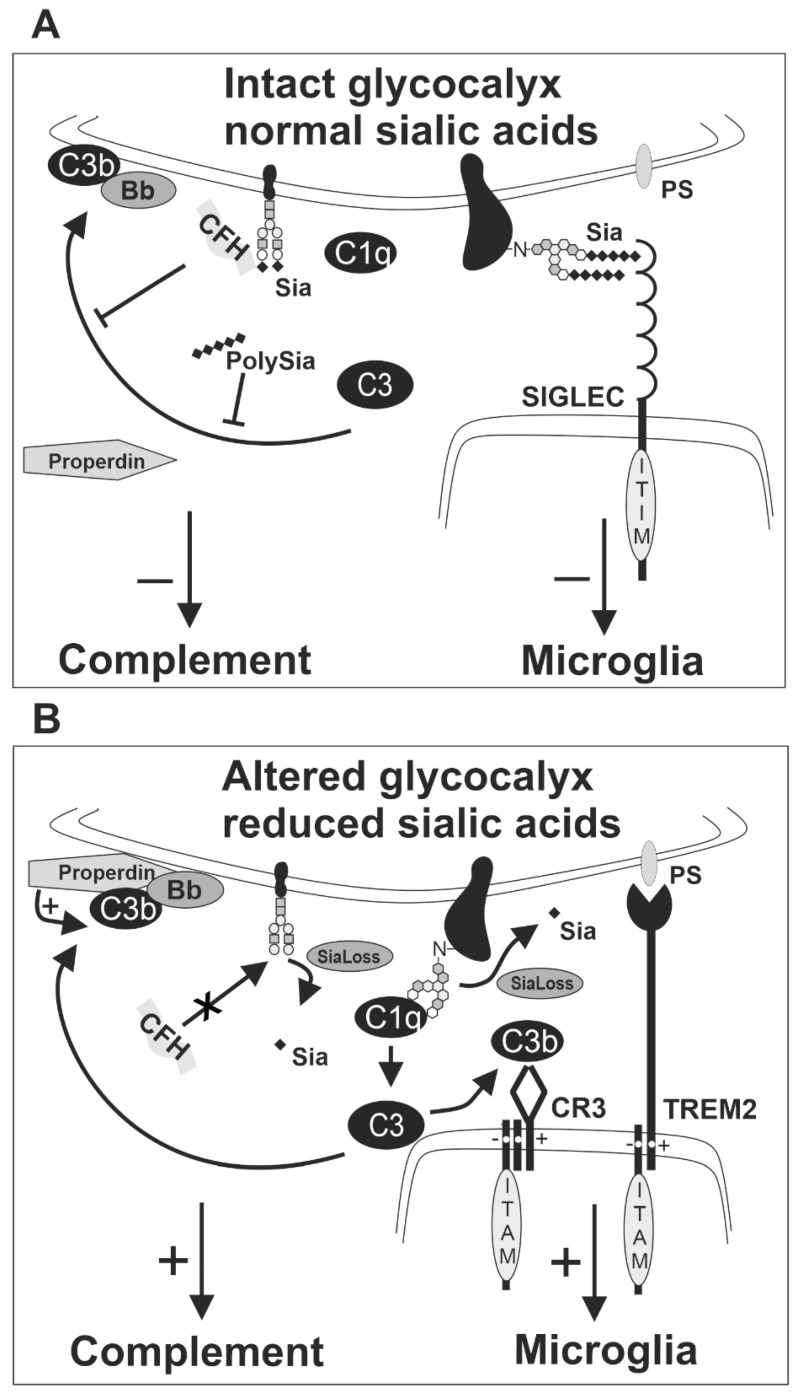
Sialic acid of the intact glycocalyx as a checkpoint for inhibition of complement and microglia. (**A**) The alternative complement pathway is triggered by spontaneous hydrolysis of C3 into the active form C3b. The regulatory complement protein complement factor H (CFH) can bind to sialic acid residues of the host cell glycocalyx and form the C3b-CFH-Sia complex under physiological conditions. Thus, CFH can inhibit the formation of the C3 convertase (C3bBb) from the active C3b by competing with complement factor B. Consequently, CFH inhibits the activation of the alternative complement pathway on cells with intact glycocalyx. Likewise, soluble polysialic acid (polySia) has been shown to inhibit the activation of the alternative complement pathway. Furthermore, SIGLEC receptors on microglia recognize sialic acids on the glycocalyx and inhibit the activatory downstream response of microglia. (**B**) Weak binding of C3b on lesioned cells leads to activation of the alternative pathway without CFH binding. Furthermore, properdin binds to lesioned cells and promotes C3b activation and the formation of the active C3 convertase (C3bBb). Thus, loss or removal of sialic acids (SiaLoss), by neuraminidases, oxidative damage or aging triggers alternative complement activation and facilitates C3b activation and formation of the C3 convertase. Active C3b could bind to CR3 of microglia and activate the microglial cells via the ITAM of TYROBP/DAP12. Furthermore, other ITAM-signaling receptors of microglia, such as TREM2, recognize aminophospholipids, such as phosphatidylserine (PS), that become externalized and accessible after enzymatic removal or loss of the negatively charged sialic acids. The activatory receptors cannot be silenced by the SIGLEC receptors that fail to sense sialic acids on the altered glycocalyx of the lesioned cell. Bb, a subunit of complement factor B; C1q, complement component 1q; C3, complement component 3; C3b, complement component 3b; CFH, complement factor H; CR3, complement receptor 3 as heterodimer of CD11b/ITGAM and CD18/ITGB2; ITAM, immunoreceptor tyrosine-based activation motif; ITIM, immunoreceptor tyrosine-based inhibition motif; polySia, polysialic acid; PS, phosphatidylserine; Sia, sialic acid; SiaLoss, desialylation; SIGLEC, sialic acid-binding immunoglobulin type lectin; TREM2, triggering receptor expressed on myeloid cells 2.

Sialic acid residues can bind CFH and thereby locally inhibit complement activation (Figure 3). A recent study demonstrated that sialylation is critical for protecting fetal extraembryonic tissue from maternal complement attack [98], although the mechanism remained unclear. The addition of free Sia monosaccharides to normal human serum decreased the level of C3 and complement component 5, but not CFH and CFI. Moreover, the addition of free Sia to human serum challenged by LPS led to less cleavage of C3 and CFB. Therefore, free Sia might negatively regulate the activation of C3 and the following complement cascade [99]. However, relatively high concentrations of free Sia were needed to see these effects, questioning whether soluble Sia is as powerful for the complement inhibition as glycan-associated Sia. Importantly, CFH only binds to α2.3-linked Neu5Ac, but not to free Sia monosaccharides [100]. When CFH binds to α2.3-linked Neu5Ac on the cellular glycoconjugates in physiological conditions, it can form the C3b-CFH-Sia complex [8]. This complex is crucial for inhibiting complement amplification [96,101]. Thus, Sias on glycoconjugates are the critical determinants for CFH–mediated complement inhibition. Recently, we found that low molecular polySia, a homopolymer of α2.8-linked Sias with an average degree of polymerization 20 (polySia avDP20), also attenuated alternative complement activation. Thereby, polySia was leading to less cell lysis and less membrane attack complex formation [89]. Since CFH binds to α2.3-linked Sia, but not to α2-6- or α2-8-linked Sia [100], the mechanism of inhibitory effects of polySia avDP20 on the alternative pathway remains elusive.

Most SIGLEC receptors contain intracellular ITIMs. Besides conserved SIGLEC-2, these SIGLECS include SIGLEC-E, -F, and -G in mice and SIGLEC-3, -5, -6, -7, -8, -9, -10, -11, and -12 in humans [102]. Upon extracellular sialoconjugate ligand binding to SIGLECS, the intracellular ITIMs counteract the activatory signal proceeding from ITAM-containing receptors [9,19,103]. The counteraction is initiated via phosphorylation of ITIMs by SRC family kinases after ligand binding. Then, the ITIM tyrosine recruits tyrosine phosphatases, such as SRC homology region 2 domain-containing phosphatase-1 (SHP-1/PTPN6) or SRC homology region 2 domain-containing phosphatase-2 (SHP-2/PTPN11) [102], which dephosphorylate the signaling molecules in the ITAM-signaling cascade to suppress the activation of the respective immune cells. Thus, immune cell-mediated activation processes such as phagocytosis, oxidative burst or inflammation are attenuated [104] (Figure 1).

Several studies have shown that both phosphatases SHP-1 and SHP-2 participate in ITIM signaling of SIGLECS [9,105,106,107,108,109,110,111]. Knock-out mice for either *Shp-1* or *Shp-2* were not viable. While knock-out of *Shp-1* resulted in premature lethality, knock-out of *Shp-2* led to embryonic lethality [112]. Thus, viable motheaten mutant mice (meV/meV) with partial loss of *Shp-1* function and conditional-knockout mice lacking *Shp-2* in defined cell types helped to investigate the function of SHP-1 and SHP-2 in the brain, respectively. In the motheaten mouse brain, reduced expression of SHP-1 resulted in decreased numbers of astrocytes, microglia, and oligodendrocytes [113]. In contrast, the absence of *Shp-2* in the oligodendrocyte lineage led to impaired generation of oligodendrocyte progenitor cells [114]. Since inhibitory SIGLECS could activate SHP1/2, reduced expression of SHP-1 might lead to over-activation of microglia. Indeed, after challenge of microglia isolated from meV/meV mice with reduced SHP-1 by LPS, more neurotoxic mediators were released [115]. Moreover, higher levels of pro-inflammatory cytokines and lower levels of anti-inflammatory cytokines were presented in the hindbrain of the meV/meV mice [116]. Furthermore, retinal degeneration was observed in the meV/meV mice with reduced SHP-1 expression [117]. Similarly, the deletion of *Shp-2* within radial glia led to increased gliogenesis and decreased neural genesis [118]. Hence, SHP-1/2 signaling plays a critical role in brain homeostasis by modulating the microglia activation status.

The ITAM-SYK-signaling pathways of TREM2-DAP12 and CR3-DAP12 are phagocytosis-related. Multiple studies have shown that ITIM signaling of SIGLECS counteracts this pathway. A previous study showed that binding of sialylated oligosaccharide ligands or monoclonal antibodies to SIGLEC-5 inhibited macrophage-mediated phagocytosis of apoptotic bodies [119]. Furthermore, SIGLEC-11 inhibited the uptake of apoptotic neuronal material by microglia and attenuated inflammation caused by LPS [120]. Moreover, mouse SIGLEC-E impaired microglial phagocytosis of neural debris and phagocytosis-associated oxidative burst [121]. Likewise, human SIGLEC-3 prevented microglial uptake of aggregated amyloid-β 42 peptide [122]. Most recently, it was reported in mice that SIGLEC-2 downregulated the microglial phagocytic ability during aging and the blockage of SIGLEC-2 promoted removal of α-synuclein fibrils, amyloid-β oligomers, and myelin debris in vivo [123].

ITIM-containing SIGLECS might also inactivate the inflammasome via inhibiting SYK signaling that was triggered by activatory ITAM-signaling receptors [124] (Figure 1). It was reported that both c-Jun NH2-terminal protein kinase (JNK) and SYK are necessary kinases for inflammasome activation [124,125]. Whereas toll-like receptor (TLR) signaling contributes to JNK kinase activation, ITAM receptor signaling stimulates SYK kinase. The inflammasome mainly participates in both pro-inflammatory and host-protective immune responses [126]. Most recently, it has been demonstrated that NACHT, LRR, and PYD domains-containing protein 3 (NALP3/NLRP3) inflammasome activation plays a vital role in the pathogenesis of tauopathies [127]. Importantly, SIGLEC-5 reduced NLRP3 inflammasome activation in response to Group B *Streptococcus* (GBS) infection [128]. Similarly, GBS β-protein bound SIGLEC-7 to inhibit NLRP3 inflammasome activation and attenuated inflammation in natural killer cells [129]. Thus, targeting the inflammasome by modulation of ITIM-containing SIGLECS might be a novel therapeutic strategy for treating neurodegeneration.

## 6. Effects of Desialylation on Brain Innate Immunity

Desialylation enables cellular responses and is associated with human physiological and pathological processes [68]. In particular, desialylation is the first step in triggering brain innate immunity. It has been shown that postnatal inflammatory exposure causes ultimate increase in the activity of the neuraminidases NEU1 and NEU4. This increased neuraminidase activity immediately resulted in desialylation of glycoproteins of neural cells [130]. Furthermore, oxidative damage has been shown to desialylate cell surfaces [131]. Removing terminal Sia of glycoconjugates by sialidases or oxidative damage then initiates a series of cell-signaling events of innate immunity.

First of all, desialylation might attenuate interaction between sialic acid residues with CFH, leading to enhanced complement cascade activation (Figure 3). Indeed, rats treated with neuraminidases had C3 activation fragments in serum and cerebrospinal fluid. Moreover, neuraminidase treatment caused complement system activation and enhanced ependymal damage [132]. Previously, it was shown that enzymatic removal of Sia from the neuronal glycocalyx of cultured neurons promoted the binding of complement protein C1q to neurites, which then triggered the removal of the neurites by cocultured microglial cells via the CR3 [41]. Most recently, it was demonstrated that activated microglia had increased surface sialidase activity and desialylated their own cell surface. This further stimulated CR3-mediated phagocytosis of neurons by microglia [133].

Additionally, desialylation modulates SIGLEC or other sialic acid binding receptor signaling. Desialylation of neurons or microglia enhanced microglial phagocytosis of neurons or neuronal parts via decreased activation of inhibitory SIGLECS, including: SIGLEC-2 (CD22) [123], SIGLEC-3 (CD33) [134], SIGLEC-11 [120], SIGLEC-E [121], and SIGLEC-F [135]. In parallel, various receptors involved in innate immunity, including TLR4, were found to be activated after desialylation [136]. Interestingly, desialylation could also increase neuronal sprouting. Interaction between gangliosides and MAG (SIGLEC-4) stabilizes myelin. However, when sialic acid residues of gangliosides were removed by sialidases, axon sprouting outgrowth was visible in rats after lesions [137,138].

Third, the loss of Sias by desialylation can change the binding of the lectin galectin-3 and the neurotrophic factor BDNF. Galectin-3 was released by LPS-stimulated microglia. When desialylation of neurons occurred, Galectin-3 bound to galactose residues of glycoproteins on neurons that normally were occupied by terminal Sia residues. This process enhanced the phagocytosis of neurons by microglia [139]. In addition, Neu1 removed polySia on microglia that were stimulated by LPS, leading to the release of BDNF that was retained by polySia [86].

Importantly, insufficient activity of sialidases/neuraminidases can also have detrimental effects. Several studies indicated that removal of glycan residues is a prerequisite for proper digestion and re-use of lipids and proteins [70,140,141]. For example, NEU3 and NEU4 are responsible for removing Sias from the gangliosides during lysosomal digestion. In the absence of both *Neu3* and *Neu4*, non-digested ganglioside GM3 accumulated in microglia, vascular pericytes and neurons. This abnormal storage further caused micro- and astrogliosis, neuroinflammation, and accumulation of lipofuscin bodies in the mouse brain [140]. Moreover, it is well-known that neuraminidase deficiencies within the lysosome cause accumulation of undegraded substrates, such as glycocalyx, leading to neurodegenerative lysosomal storage diseases. For example, genetic deficiency of *NEU1* or reduced levels of NEU1 caused by a genetic defect of Cathepsin A in humans cause sialidosis and result in galactosialidosis with lysosomal accumulations and neurodegeneration [70]. In a genetic mouse model for galactosialidosis, microglia and perivascular macrophages showed extensive carbohydrate macromolecule accumulation in consequence of this lysosomal storage dysfunction, while neurons were affected to a lesser extent [141]. Thus, neuraminidase deficiencies could lead to accumulation of non-digested glycolipids in lysosomes of mononuclear phagocytes, which might interfere with their function. Particularly, these lipid-laden macrophages and microglia possibly fail to clear aggregates or trigger inflammatory neurodegeneration.

Of note, the amyloid precursor protein (APP) is a natural substrate of NEU1. In the *Neu1* KO mice, APP was over-sialylated and accumulated in the lysosomes of the neural tissue. As a consequence of this lysosomal accumulation in neurons, excessive lysosomal exocytosis in the extracellular space of amyloid-β peptides was triggered [142]. Moreover, *Neu1* ablation in an AD mouse model overexpressing the human mutant APP accelerated amyloid-β production, whereas *NEU1* overexpression by injection of an adeno-associated virus containing human *NEU1* reversed the increased amyloid-β plaque load [142]. Hence, reduced NEU1 activity may be a risk factor for developing AD and targeting NEU1 could be a potential therapeutic option.

Similarly, multiple mouse models of lysosomal storage diseases present a neurodegenerative pathology [143,144]. Dysfunctional microglia related to impaired activity of lysosomes or phagosomes might be the major trigger of the pathology in lysosomal storage disease. It has been shown that the accumulation of Sia in endocytic compartments inhibits the maturation of lysosomes, leading to impaired functions of the lysosomal system [145]. Furthermore, Sias on *Pseudomonas aeruginosa* indirectly inhibited phagosome maturation in macrophages. Possibly, this process was caused by reduced phagosome lysosome fusion [146]. Importantly, lysosomes contribute to the final steps of both phagocytosis and autophagy via degradation and recycling of extra- and intra-cellular substances. Thus, delocalized Sia might prevent maturation of lysosomes and phagosomes with potential consequences for diseases requiring lysosomal degradation of mis-folded proteins or digestion of glycolipids.

## 7. Sia-Complement Axis and Sia-SIGLEC Axis in Brain Disorders

Synapse loss is a major pathological feature in many neurodegenerative diseases, including AD [147]. Previous research suggested that inappropriate over-activation of the classical complement cascade mediates synaptic elimination by microglial phagocytosis early in AD animal models [40,148]. Intriguingly, synaptic loss often occurs before the loss of neurons in neurodegenerative diseases [149,150]. As mentioned above, in vitro studies have indicated that the removal of neurites by microglia could be mimicked by desialylation of neurons or microglia [41,42,133]. When neurons were desialylated, C1q bound to neurites and the opsonized structures were engulfed via CR3 into the co-cultured microglia [41,42]. Likewise, the cell-autonomous desialylation of microglia after their activation stimulated CR3-mediated phagocytosis of neurons in a co-culture system [133].

To further investigate the role of partial desialylation in the brain, we recently analyzed viable heterozygous *Gne* knockout mice that have slightly (~20–30%) reduced sialylation levels [90]. Of note, homozygous *Gne* knockout in mice is embryonically lethal [151]. In these viable heterozygous *Gne* knockout mice, microglial cells were activated and showed reduced arborization at 6–9 months of age. In addition, synapse numbers were reduced, followed by a gradually increased neuronal loss at 12 months of age. Furthermore, crossbreeding these mice with complement C3-deficient mice showed that not only the loss of neurons and synapses, but also the reduced microglial arborization were dependent on complement C3 [90]. These data indicated that also in vivo the Sia-complement axis plays a critical role in modulating microglial phagocytosis in both aging and neurodegenerative diseases.

Both neurodegeneration and aging have been associated with dysfunction or deletion of SIGLECS [13,102,152]. SIGLECS on microglial cells interact with sialic acid residues to inhibit microglial activation, inflammation, phagocytosis, and oxidative burst. Since Sias are ubiquitously present on most neural cells, SIGLECS of microglia could interact in *cis* and *trans* [9]. While *trans* interaction means that SIGLECS bind to Sias on the cell surface of neighboring cells, *cis* interaction means that SIGLECS interact with Sias on the surface of the same cell. While all SIGLECS expressed on microglia including mouse SIGLEC-2, human SIGLEC-3, SIGLEC-11, mouse SIGLEC-E, and SIGLEC-F were shown to have *trans* interactions with Sias of glycoconjugates on neighboring cells [102,109,121,123,153], only human SIGLEC-3 and SIGLEC-E have been demonstrated so far to also bind Sias of glycoconjugates in *cis* [121,154]. The microglial human SIGLEC-3, SIGLEC-11 and mouse SIGLEC-E show inhibitory signaling.

Human SIGLEC-11 was first studied functionally by ectopic expression on murine microglia [120]. It turned out that SIGLEC-11 downregulated proinflammatory mediators in LPS-challenged microglia. SIGLEC-11 also impaired microglial phagocytosis of apoptotic neuronal material. This neuroprotective effect of SIGLEC-11 was mediated by *trans* interaction with polySia on neurons, but not *cis* interaction [120].

Similarly, SIGLEC-E also has a neuroprotective effect. We found that SIGLEC-E inhibited phagocytosis of neural debris [121]. Moreover, while neural debris triggered superoxide release and production of proinflammatory cytokines, intact SIGLEC-E on microglia prevented this activation phenotype. Data indicated that the neuroprotective role of SIGLEC-E was mediated via recognition of neuronal Sia residues, although SIGLEC-E also has a lower extent of *cis* interaction [121]. In addition, neuraminidase-treated neurons reduced binding with SIGLEC-F on microglia and this Sia–SIGLEC-F interaction protected the integrity of neurons [153].

As mentioned above, human SIGLEC-3 (also known as CD33, which is abundantly expressed on microglia) is thought to have both *trans* and *cis* interaction with Sia of glycoconjugates. It generates two splice variants with relevance for AD. The full-length CD33M isoform has an intact exon 2, which encodes the Sia ligand-binding domain [155]. Moreover, the full-length CD33M isoform showed increased expression in microglial cells in AD brains [122,155] and turned out to be a risk factor for AD in several genome-wide association studies [156,157,158,159]. Oppositely, the shorter CD33m isoform without exon 2 was associated with a decreased AD risk [134,155,160,161,162]. Interestingly, increasing human CD33M levels prevented microglial uptake of amyloid-β 42, whereas inactivation of CD33 attenuated amyloid-β pathology in an AD mouse model system, possibly via higher uptake of amyloid-β 42 [122]. Thus, the weakening of the Sia-SIGLEC-3 axis via desialylation or variants of SIGLEC-3/CD33m with a lack of the Sia ligand-binding domain might increase microglial phagocytosis, thus resulting in alleviated AD pathology.

SIGLEC-2, also called CD22, has been claimed to be upregulated in microglia during aging [123]. In addition to aging, multiple human RNA-seq datasets suggested upregulation of *SIGLEC-2* in the brains of AD patients [163]. Similarly, *SIGLEC-2* is upregulated in brains with other neurodegenerative disorders [164,165]. Importantly, SIGLEC-2 negatively regulated microglial phagocytosis during aging in mice [123]. SIGLEC-2 on microglia bound to synthetic glycoconjugates bearing α2–6-linked Sia. This interaction mediated an anti-phagocytic effect of microglia. Of note, inhibition of SIGLEC-2 with SIGLEC-2 blocking antibody or genetic ablation enhanced removal of amyloid-β oligomers, myelin debris and α-synuclein fibrils in mice. Strikingly, long-term SIGLEC-2 blockade via CNS-delivery of an antibody blocking SIGLEC-2 function restored microglial homeostasis and attenuated cognition deficits in aged mice [123]. However, it is unclear whether SIGLEC-2 of microglia also interacts in *cis* with Sia.

## 8. Conclusions

Sias of glycoconjugates play a critical role in controlling innate immunity of the nervous tissue. Accumulating evidence shows that the innate immune system senses Sias of glycoconjugates via several receptors to maintain homeostasis of the nervous tissue, and only responds appropriately in a very local and time-restricted manner. The existence of Sia is critical for preventing neural tissue damage via interaction with the complement system and SIGLEC receptors. Thus, well-defined regulation of sialylation is essential for the nervous tissue. Interestingly, this Sia checkpoint allows even subcellular responses of microglia, e.g., phagocytosis signaling at the site of a single cellular process sensing and touching a protein aggregate.

Based on these facts, modulating the Sia status on glycoconjugates could be a potential therapeutic strategy for treating neuroinflammatory disorders and preventing age-related inflammatory processes. Principally, therapeutic effects could be realized at different levels, namely by regulating sialyltransferases, supplementation of Sia-linked oligosaccharides/polySia or by regulating extracellular sialidases.

Our recent data showed that the local application of polySia avDP20 is a potential candidate for treating inflammatory processes of the retina, since polySia avDP20 showed anti-inflammatory and anti-oxidative effects on cultured *SIGLEC-11* expressing human mononuclear phagocytes in vitro [166]. Furthermore, intravitreal application of polySia avDP20 in the vitreous body of humanized *SIGLEC-11*-transgenic mice inhibited the reactivity of mononuclear phagocytes, blocked the complement system and prevented the retinal lesion and vascular damage induced by laser coagulation [89]. Thus, the applied polySia avDP20 strengthened the ‘Sia-SIGLEC’ and the ‘Sia-complement’ axes and was beneficial for the retinal tissue.

Hence, targeting the Sia checkpoint might be a promising therapeutic approach to treat age-related neurodegenerative diseases. However, there are several caveats. Exploring the roles of SIGLEC and the complement system of humans by only employing mouse models should be avoided. Due to the rapid evolution of human SIGLECS, human and murine have different expression and ligand-binding profiles. Furthermore, only a few SIGLECS (SIGLEC-1/-2/-4/-15) are highly homologous between humans and mice, while most other SIGLECS have no strict homologs in structure and function in other species [102]. Of note, human and mouse CD33 have the same name, but appear to be quite different since human CD33 has an ITIM and an ITIM-like domain, while mouse CD33 solely has an ITIM-like domain [167]. Accordingly, human and mouse CD33 show different functions [168]. Human CD33 inhibited monocyte and microglial phagocytosis, whereas mouse CD33 had no effect on phagocytosis [168]. Likewise, humans have complement receptor CR1 without a direct homolog in mice, while mice have the receptor Crry that acts as regulator of complement C3, without a homolog in humans [169]. In addition, humans have five CFH-related proteins (CFHR1, CFHR2, CFHR3, CFHR4, and CFHR5) based on their genes, while mice have fewer CFH-related genes. So far, only the expression of factor H-related protein B and C (FHR-B and FHR-C) was observed in mice [170,171].

In addition, Sia composition is tremendously different between humans and other mammals. Humans can only produce the Sia form Neu5Ac due to a loss-of-function mutation in the enzyme cytidine monophospho-N-acetylneuraminic acid hydroxylase [172], while most other mammals including mice synthesize and express Neu5Ac and Neu5Gc [173]. Notably, the expression of Neu5Gc in non-human vertebrate brains is either undetectable or present only at very low levels, even in mammals, in which Neu5Gc is the major Sia of most tissues [174]. However, the consequences of accidentally incorporated Neu5Gc in brains remain elusive. Therefore, it still needs to be determined whether Neu5Gc can be detected in the brains of humans under disease conditions and how any incorporated Neu5Gc interferes with the function of the brain.

Finally, it is still unclear in which diseases and at which disease stage the ‘Sia-SIGLEC’ and/or ‘Sia-complement’ axes should be strengthened or weakened. While several data indicate that a weakening of the axes might help to clear amyloid-β plaques in AD animal model, the overall concept suggests to strengthen both axes and thereby avoid overt complement- and radical-mediated damage of postmitotic neuronal cells. More importantly, it is fully obscure whether there is any contribution of the non-human sialic acid type Neu5Gc to the accumulation of lipids and proteins in lysosomes, since humans can incorporate Neu5Gc by eating red meat and dairy products [175,176], but cannot properly digest Neu5Gc by their endogenous neuraminidases after incorporation in α2-8-linked oligo- and polysialic acids [174]. Furthermore, incorporated Neu5Gc triggers auto-antibodies in all humans described as ‘xenosialitis’ with unknown pathogenicity [177].

## Abbreviations

AD	Alzheimer’s disease
APP	Amyloid precursor protein
Bb	A subunit of complement factor B
BDNF	Brain-derived neurotrophic factor
C1	Complement component 1
C1q	Complement component 1q
C3	Complement component 3
C3a	Complement component 3a
C3b	Complement component 3b
CFB	Complement factor B
CFH	Complement factor H
CFI	Complement factor I
CMP	Cytidine 5’-monophospho
CNS	Central nervous system
CR3	Complement receptor 3
DAM	Disease-associated microglia
*E. coli* K1	*Escherichia coli* K1
Fuc	Fucose
Gal	Galactose
GalNAc	N-acetylgalactosamine
GBS	Group B *Streptococcus*
Glc	Glucose
GlcNAc	N-acetylglucosamine
GNE	Glucosamine (UDP-N-Acetyl)-2-epimerase/N-acetylmannosamine kinase
HSCT/DAP10	Hematopoietic cell signal transducer
IL	Interleukin
ITAM	Immunoreceptor tyrosine-based activation motif
ITIM	Immuno-receptor tyrosine-based inhibitory motif
JNK	C-Jun NH2-terminal protein kinase
Kdn	Deaminoneuraminic acid
LPS	Lipopolysaccharides
MAG	Myelin-associated glycoprotein
Man	Mannose
ManNAc	N-Acetyl-D-mannosamine
ManNAc-6-P	N-Acetyl-mannosamine 6-phosphate
NADPH	Nicotinamide adenine dinucleotide phosphate
NALP3/NLRP3	NACHT, LRR and PYD domains-containing protein 3
NCAM	Neural cell adhesion molecule
NEU1	N-acetyl-alpha-neuramindase 1
NEU2	N-acetyl-alpha-neuraminidase 2
NEU3	N-acetyl-alpha-neuraminidase 3
NEU4	N-acetyl-alpha-neuraminidase 4
Neu5Ac	N-acetylneuraminic acid
Neu5Gc	N-glycolylneuraminic acid
P	Phosphate
polySia avDP20	Polysialic acid homopolymer with an average degree of polymerization of 20
polySia	Polysialic acid
PS	Phosphatidylserine
RNA-seq	RNA sequencing
ROS	Reactive oxygen species
SHP-1	Src homology region 2 domain-containing phosphatase-1
SHP-2	Src homology region 2 domain-containing phosphatase-2
Sia	Sialic acid
SiaLoss	Desialylation
SIGLEC	Sialic acid-binding immunoglobulin-like lectin
SIRP	Signal regulartory protein
SYK	Spleen tyrosine kinase
TLR	Toll-like receptor
TREM2	Triggering Receptor Expressed on Myeloid Cells 2
TYROBP/DAP12	TYRO protein tyrosine kinase binding protein/DNAX-activating protein of 12 kDa
